# Distinct changes to pancreatic volume rather than pancreatic autoantibody positivity: insights into immune checkpoint inhibitors induced diabetes mellitus

**DOI:** 10.1186/s13098-024-01263-6

**Published:** 2024-01-23

**Authors:** Hung-Hui Wei, Ying-Chieh Lai, Gigin Lin, Cheng-Wei Lin, Ya-Chu Chang, John Wen-Cheng Chang, Miaw-Jene Liou, I-Wen Chen

**Affiliations:** 1 Division of Endocrinology and Metabolism, Chang Gung Memorial Hospital at Linkou, 5, Fusing St., Guishan Dist, Taoyuan City, 333 Taiwan; 2 Department of Medical Imaging and Intervention, Chang Gung Memorial Hospital at Linkou, Taoyuan City, Taiwan; 3 Division of Hematology-Oncology, Chang Gung Memorial Hospital at Linkou, Taoyuan City, Taiwan; 4 College of Medicine, Chang Gung University, Taoyuan City, Taiwan; 5 Department of Medical Imaging and Radiological Sciences, Chang Gung University, Taoyuan, Taiwan; 6 Clinical Metabolomics Core, Chang Gung Memorial Hospital at Linkou, Taoyuan, Taiwan; 7 Institute for Radiological Research, Chang Gung University, Taoyuan, Taiwan; 8 Immuno-Oncology Center of Excellence, Chang Gung Memorial Hospital at Linkou, Taoyuan, Taiwan

**Keywords:** Immune check point inhibitors, Autoimmunity, Diabetes mellitus, Insulin-dependent-diabetes, Pancreatic volume

## Abstract

**Background:**

Immune checkpoint inhibitors (ICI) are promising treatment options for various cancers. However, their use is associated with immune-related adverse events (irAEs), including ICI-induced diabetes mellitus (ICI-DM). This study aimed to investigate the clinical features of ICI-DM, with a particular focus on alterations to pancreatic volume.

**Methods:**

We conducted a retrospective review of 2829 patients who received ICI treatment at the Chang Gung Memorial Hospital, Linkou, between January 2014 and December 2021. New-onset diabetes or diabetic ketoacidosis (DKA) was identified in ten patients receiving ICI therapy. Pancreatic volumes were assessed by manual segmentation of computed tomography (CT) images before and after ICI-DM diagnosis.

**Results:**

Among these ten patients, nivolumab was the most commonly used ICI (50.0%), followed by pembrolizumab (30.0%) and atezolizumab (20.0%). One patient received combination therapy with nivolumab and ipilimumab. The median age was 63.01 years (range: 40.1 − 87.8). ICI-DM developed after a median of 13.5 cycles (range: 2 − 42) of ICI treatment or 9.85 months (range:1.5 − 21.3) since ICI initiation. The initial presentation was DKA in 60.0% of patients. All patients had low or undetectable C-peptide levels (range: <0.033 − 0.133 nmol/L) and were negative for most type 1 diabetes mellitus (T1DM)-related autoantibodies; only one patient tested positive for glutamic acid decarboxylase antibodies. CT imaging revealed significant pancreatic atrophy, with a median pancreatic volume decrease of 19.92% (*P* = 0.038) from baseline and sustained significant decline at last follow-up (median − 37.14%, *P* = 0.012).

**Conclusions:**

ICI-DM is often accompanied by pancreatic atrophy and approximately two-thirds of patients initially present with DKA. Although the majority of ICI-DM patients lack T1DM-related autoantibodies, identifying diminished pancreatic volumes through CT imaging provides valuable clues into the subclinical aspects of ICI-DM development, aiding in the prevention of diabetic emergencies.

**Trial registration:**

Not applicable.

**Supplementary Information:**

The online version contains supplementary material available at 10.1186/s13098-024-01263-6.

## Background

Recently, immune checkpoint inhibitors (ICI), which include monoclonal antibody blockade of the cytotoxic T-lymphocyte antigen 4 (CTLA-4), programmed cell death 1 (PD-1) receptor or its ligand (PD-L1), have emerged as a widely used treatment option for various cancer types [1]. While this therapy has shown promise, it can induce immune-related adverse events (irAEs) due to the exaggerated immune response towards non-cancerous cells, resulting in significant inflammation and destruction [2]. The most commonly affected organ systems are the skin, gastrointestinal system and liver, and the endocrine system [3, 4]. In terms of irAEs, endocrine disruptions are the most prevalent and irreversible, requiring lifelong hormone replacement and can lead to hospitalization or death if left undetected or untreated.

One rare but potentially life-threatening irAE is ICI-induced diabetes mellitus (ICI-DM), which occurs in approximately 0.86 − 1.27% of patients receiving ICI therapy. The incidence of ICI-DM increases as ICIs become more widely used [1, 5, 6]. Anti-PD-1, anti-PD-L1, and anti-CTLA-4 therapies have reported ICI-DM incidences of 1.18%, 0.73%, and 0.33%, respectively; a higher rate of up to 2.60% is reported for anti–CTLA-4/anti–PD-1/anti–PD-L1 combination therapy [1]. Among patients diagnosed with ICI-DM, 30 − 76% initially present with diabetic ketoacidosis (DKA), and almost all require hospitalization and lifelong insulin therapy [6, 7]. Unlike type 1 diabetes mellitus (T1DM), the onset of ICI-DM appears to be more abrupt, and T1DM autoantibody positivity is less frequent, approximately occurring in only half the cases [8]. Glutamic acid decarboxylase antibodies (GAD-Ab) are present in over 80% of T1DM patients by the time of diagnosis, [9, 10] but their presence is less common in ICI-DM patients. Since the introduction of ICIs, several fulminant type 1 diabetes mellitus (FT1DM) case reports have been published [11, 12]. FT1DM is characterized by marked hyperglycemia and a near-normal level of glycated hemoglobin (HbA1c) despite marked hyperglycemia, ketoacidosis, negative autoantibodies against pancreatic β cells, severe insulin deficiency, and elevated pancreatic enzyme levels [13].

The pancreas plays a critical role in regulating blood glucose levels, and changes to its volume and structure can affect its functionality. Generally, pancreatic volume increases linearly with age during childhood and adolescence, reaches a plateau from age 20 to 60 years, and then declines thereafter [14]. Most studies have found that the pancreatic volume is smaller in patients with diabetes [15]. Despite the well-established link between pancreatic volume and diabetes, there is limited data on the imaging characteristics of the pancreas in ICI-DM [16, 17]. Therefore, it is crucial to understand the imaging characteristics of the pancreas in patients with ICI-DM to identify potential diagnostic clues that could aid the early detection and management of this uncommon irAE.

We conducted the largest single-institution case series in Taiwan to better understand clinical features associated with the uncommon irAE, ICI-DM. In this study, we aimed to identify key findings that could potentially serve as clinical and diagnostic clues, including imaging characteristics of the pancreas in patients with ICI-DM.

## Methods

### Participants and case definition

This retrospective study analyzed patients who received CTLA-4, PD-1, or PD-L1 inhibitors for cancer treatment at Linkou Chang Gung Memorial Hospital from January 2014 to December 2021. This study was approved by the Institutional Review Board of the Chang Gung Memorial Hospital (No. 202201766B0). To define ICI-DM in this case series, we used the following criteria:

1. New onset of hyperglycemia that required exogenous insulin treatment in patients who:
  A. Had no history of diabetes or
  B. Had a history of type 2 diabetes mellitus (T2DM) but became insulin-dependent without an attributable cause and showed deterioration in glycemic control, which was previously well controlled with oral medications alone.
2. Continued insulin requirement for more than 1 month with evidence of insulin deficiency, either through presentation with DKA or low or absent random C-peptide levels.

We modified the diagnostic criteria for FT1DM set by the Japan Diabetes Society [18] according to Angelos et al., [11] taking into consideration the relatively low prevalence of ICI-DM and heterogeneity in the diagnostic workup. To be diagnosed with FT1DM in the context of ICI-DM, the following criteria had to be met: occurrence of DKA (approximately 7 days) after the onset of hyperglycemic symptoms, plasma glucose level ≥ 16.0 mmol/L and HbA1c < 72 mmol/mol at the first visit, and fasting serum C-peptide level < 0.1 nmol/L.

Patients on glucocorticoids, pre-existing T1DM, T2DM on regular insulin used during ICI, or pancreatic metastasis were excluded. Baseline variables were collected at the start of ICI therapy and diabetes-related clinical and laboratory variables were collected at the time of diagnosis and at the most recent follow-up.

### Imaging evaluation and laboratory testing

Computed tomography (CT) was conducted at the Department of Radiology at Linkou Chang Gung Memorial Hospital and interpreted by two experienced radiologists. Pancreatic volumes were manually segmented from CT or MRI scans using open-source DICOM viewer (Horos Project). Volumes were calculated at four time points: the most recent CT scan prior to ICI initiation was used as the baseline, the last CT scan available before diabetes onset and immediately after ICI-DM diagnosis were used as the second and third time points, and the last CT scan available after diabetes onset was used as the final time point for comparison. Tumor assessment images from CT were reviewed again and standardized based on the Response Evaluation Criteria in Solid Tumors (RECIST) version 1.1.

The presence of autoantibodies was evaluated at the onset of diabetes and one year after diagnosis in the clinical laboratories at Linkou Chang Gung Memorial Hospital. Antibody levels for glutamic acid decarboxylase (GAD-Ab), islet antigen 2 (IA2-Ab), zinc transporter 8 (ZnT8-Ab), and insulin (IAA) were measured. GAD-Ab were measured using ELISA kits (ElisaRSR before 2020 and EUROIMMUN after 2020), whereas IA2-Ab and ZnT8 antibodies were tested using ELISA kits (ElisaRSR). IAA levels were evaluated using a radioimmunoassay (Cisbio).

### Statistical analysis

Descriptive statistics are presented as medians or ranges for continuous variables and as n (%) for nominal variables. Baseline characteristics among patients developing ICI-DM or not were compared using the Mann–Whitney U test for continuous categorical variables or the χ2 test for categorical variables. Wilcoxon rank-sum test was used for pairwise comparisons of interval pancreatic volume. Statistical significance was set at *P* < 0.05 for all analyses. All the data analyses were performed using SPSS version 26 (IBM Corp., Armonk, NY, USA).

## Results

### Incidence of ICI-DM and ICI therapy

Among the 2829 patients who received ICIs during this period, we identified 180 patients with a history of diabetes mellitus or newly diagnosed diabetes mellitus during ICI therapy, based on a review of medical charts and laboratory examinations. These patients met the criteria for diabetes according to the 2022 ADA guideline [18]. Only ten patients developed ICI-DM during the course of treatment, as detailed in Table 1. The prevalence of ICI-DM was approximately 0.35%. Of the ten patients who met the criteria for ICI-DM, nine developed new-onset insulin-dependent diabetes, while one had pre-existing T2DM that became insulin-dependent.

**Table 1:** Characteristics of the patients with ICI-induced diabetes

Patient	1	2	3	4	5	6	7	8	9	10
Age (years)	74	60	67	64	66	88	62	61	54	40
Sex	M	F	M	M	M	F	M	M	M	M
Underlying cancer	Lung CA	Ovarian CA	Melanoma	RCC	Melanoma	Melanoma	Lung CA	UC	HCC	Hard palate cancer
Type of ICI	Nivolumab	Pembrolizumab	Ipilimumab + Nivolumab	Nivolumab	Nivolumab	Nivolumab	Pembrolizumab	Atezolizumab	Atezolizumab	Pembrolizumab
Previous history of diabetes	-	-	-	-	Type 2 diabetes	-	-	-	-	-
Time to diagnosis ICI-induced diabetes after starting ICI (Onset in month)	19.6	3.8	10.7	9.0	1.5	5.0	19.8	18.1	8.9	21.3
Cycles of ICI at diagnosis ICI-induced diabetes	42	2	20	18	3	9	20	24	3	6
DKA	+	-	+	+	-	+	+	-	+	-
Glucose (mmol/L)	70.9	29.6	27.5	70.8	57.3	55.2	20.2	38.8	50.5	56.9
HbA1C (mmol/mol)	85	46	98	84	60	62	61	63	101	66
Random C-peptide (nmol/L)	0.05	< 0.03	0.06	0.04	0.13	0.08	< 0.03	< 0.03	0.09	0.06
Lipase (U/L)	14	NA	NA	376	51	11	28	NA	NA	NA
Insulin Ab (%)	4.6	6.1	7.1	7.1	5.5	NA	7.2	NA	4.6	NA
Anti-GAD* (U/mL, IU/mL)	0.18	0.12	< 0.11	< 0.57	< 0.57	< 0.57	< 0.57	< 0.57	< 0.57	624
Anti-IA2 (U/mL)	< 0.95	< 0.95	< 0.95	< 0.95	< 0.95	< 0.95	< 0.95	< 0.95	< 0.95	< 0.95
Tumor response (By RECIST)	SD	PR	PD	SD	PD	PR	SD	SD	PD	SD
Other irAE	Nil	Nil	Thyroiditis	Gastroenteritis	Nil	Nil	Colitis	Skin	Nil	Thyroiditis
ICI therapy after DKA	Stop	Stop	Continue	Continue	Continue	Stop	Continue	Continue	Stop	Stop

ICI, Immune checkpoint inhibitor; CA, carcinoma; RCC, renal cell carcinoma; UC, urothelial carcinoma; HCC, hepatocellular carcinoma; DKA, diabetic ketoacidosis; HbA1c, glycated hemoglobulin; NA, not available; Ab, antibody; GAD, glutamic acid decarboxylase; IA2, islet antigen 2; RECIST, Response Evaluation Criteria in Solid Tumors; SD, stable disease; PR, partial response; PD, progressive disease; irAE: immunotherapy related adverse effect

^*^GAD antibodies were tested with ElisaRSR before 2020, with a positive value defined as ≥ 5 U/mL; EUROIMMUN after 2020, with a positive value defined as ≥ 10 IU/mL

### Comparative analysis of patient characteristics with and without ICI-DM

After excluding patients under 18 years old, we compared the baseline clinical characteristics of 10 patients diagnosed with newly onset ICI-DM to those of 2808 patients who did not develop ICI-DM. Among the patients with newly diagnosed ICI-DM, the median age was 63.01 years (range: 40.1 to 87.8 years old), and this did not differ significantly from those without ICI-DM, whose median age was 60.61 years (range: 20.67 to 100.8 years old) (*P* = 0.559). Moreover, no significant differences were observed in gender (male in ICI-DM vs. non-ICI-DM: 80.0% vs. 59.2%, *P* = 0.182), body mass index (BMI) (ICI-DM with a median BMI of 24.08 kg/m² vs. non-ICI-DM with a median BMI of 22.93 kg/m², *P* = 0.134), and the prevalence of pre-existing diabetes mellitus (ICI-DM: 20% vs. non-ICI-DM: 19.5%, *P* = 0.267).

### Primary cancers and treatment regimens among patients with ICI-DM

Among the ten patients diagnosed with ICI-DM, melanoma (n = 3) was the most common primary cancer, followed by lung cancer (n = 2). Five patients received nivolumab, three received pembrolizumab, two received atezolizumab, and two received combination therapy with nivolumab and ipilimumab.

### Clinical and laboratory characteristics of patients with ICI-DM at presentation

The median age of ICI-DM onset was 63.01 years old (range: 40.1–87.8 years old). New-onset ICI-DM developed after a median of 13.5 cycles (range: 2–42 cycles) of ICIs or 9.85 months (range: 1.5–21.3 months) since ICI initiation. Six patients (60.0%) had an initial presentation with DKA, with a median initial glucose of 52.84 mmol/L (range: 20.2–70.9 mmol/L). The median glucose level before ICI-DM presentation was 5.38 mmol/L (range: 4.8–14.8 mmol/L), with the median value collected 25.5 days before ICI-DM (range: 14– 205 days). The median HbA1c at ICI-DM diagnosis was 64.5 mmol/mol, (range: 46–101 mmol/mol).

One of the patients (case 6) had maintained normal glucose levels from week 0 to week 16 after initiating ICI treatment. However, at 19 weeks after ICI initiation, she experienced a sudden onset of symptoms and presented with DKA, accompanied by a significant increase in blood sugar to 55.2 mmol/L. Her HbA1c at the time of diabetes diagnosis was 62 mmol/mol, indicating an acute onset of the condition (Supplementary Fig. 1).

All patients had low or undetectable C-peptide levels at ICI-DM diagnosis (range: <0.033–0.133 nmol/L). Five patients had pancreatic enzyme levels measured at the time of diagnosis, and one (20.0%) had elevated pancreatic enzyme levels.

We compared the demographic, laboratory, and clinical parameters of patients with ICI-DM to the diagnostic criteria for FT1DM established by the Japan Diabetes Society. Of the ten cases, 20.0% (2/10) fulfilled all three modified diagnostic criteria for FT1DM by the Japanese Diabetes Society, with 60.0%, 60.0%, and 90.0% meeting each criterion, respectively.

All patients were treated with insulin, and subsequently required basal and bolus regimens for diabetic control. Disease control rate was 70.0%, with 20.0% (2/10) achieving a partial response and 50.0% (5/10) maintaining stable disease. Disease progression occurred in three of the ten (30.0%) patients. Five patients discontinued ICIs after ICI-DM, although two had partial remission and two had stable disease after treatment.

Five patients (50.0%) experienced other irAEs: two with thyroiditis, one with colitis, one with gastroenteritis, and one with dermatological toxicity.

### Immunologic features

All ten patients had at least two autoantibodies associated with T1DM were measured, and only one tested positive for GAD autoantibodies. Other antibodies tested, including IA-2, ZnT8 or IAA, were negative. Three patients (cases 3, 7, and 9) were followed up for three autoantibodies, GAD-Ab, IA2-Ab, and ZnT8-Ab one year after diabetes diagnosis, all of which were negative.

### Pancreas morphology and tumor response by computed tomography

**Fig. 1:**
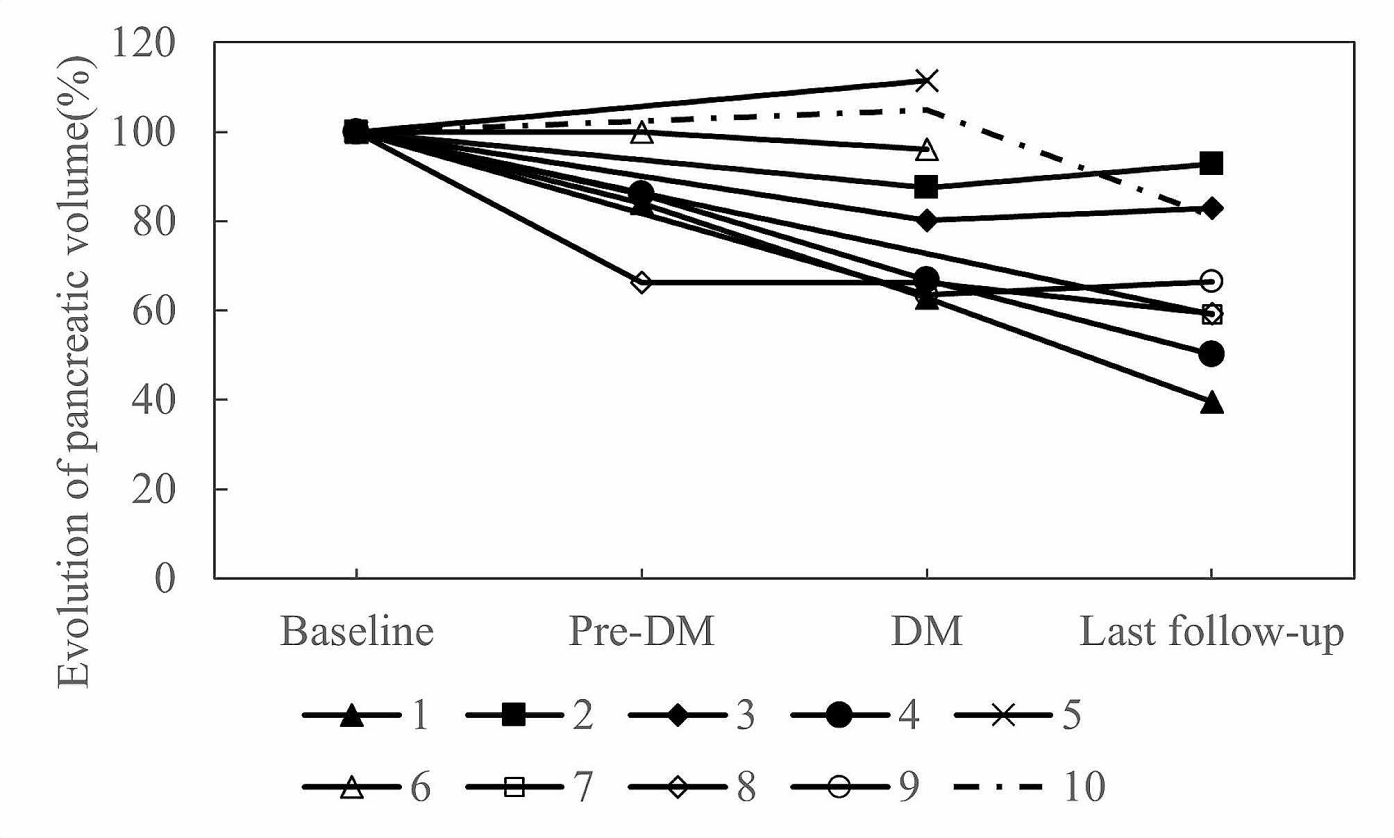
Pancreatic volume changes before and after ICI therapy. Pancreatic volume was measured using CT imaging before ICI initiation as baseline (baseline), on the last CT scan available before diabetes onset (pre-DM), immediately after diabetes onset (DM), and on the last CT scan available after diabetes onset (last follow-up) in patients with ICI-DM. Each line represents one of the ten patients included in the study

Pancreatic imaging was conducted in all ten patients, which revealed significant pancreatic atrophy following ICI-DM (Fig. 1; Table 2). None of the patients exhibited clinical signs of pancreatic exocrine insufficiency. At baseline, the median pancreatic volume was 75.80 cm^3^ (range: 44.97 − 112.83 cm^3^). Immediately after the ICI-DM diagnosis, the median pancreas volume significantly declined to 63.86 cm^3^ (range: 33.92 − 84.34 cm^3^, *P* = 0.038). Pancreatic atrophy persisted at the last available follow-up, which had a median duration of 1077.0 days (range: 473 − 2226 days), with a median pancreas volume of 55.60 cm^3^ (range: 21.38 − 74.84 cm^3^, *P* = 0.012). The pancreatic volume decreased by a median of 19.92% (range: 11.42 − -37.17%) after ICI-DM diagnosis and by 37.14% (range: -7.13 − -60.39%) at the last available follow-up image relative to baseline.

**Table 2:** Pancreatic parenchymal volumes were estimated in all ten patients by two single board-certified radiologists. Pancreatic volumes were calculated from CT or MRI scans at four time points, including before immunotherapy initiation (baseline), at the last image available before ICI-DM onset (pre-DM), at ICI-DM onset (DM), and at the last image available after diabetes onset (last follow-up)

Time of Pancreas Volume Measurement	Median (Range), cm^3^	Wilcoxon Rank Sum Test p Value	Median Percent Change (Range)
Baseline	75.80 (44.97, 112.83)	Reference	Reference
Pre-DM	60.01 (44.95, 91.99)	0.068	-14.99% (-33.84%, -0.04%)
DM	63.86 (33.92, 84.34)	0.038 *	-19.92% (-37.17%, + 11.42%)
Last follow-up	55.60 (21.38, 74.84)	0.012 *	-37.14% (-60.39%, -7.13%)

**Fig. 2:**
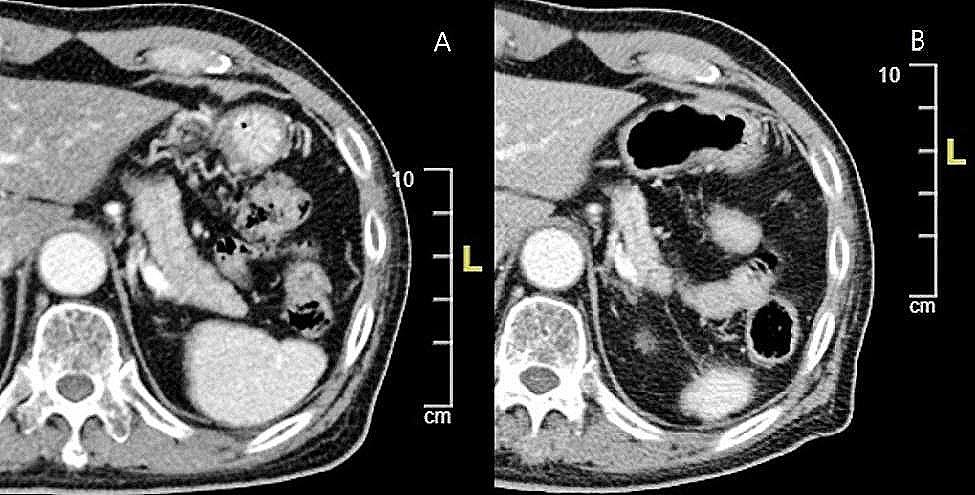
CT images of pancreas obtained before and after ICI-DM in patient 1, a patient in their 70s with lung adenocarcinoma who received anti-PD-1 therapy. (**A**) Pre-treatment CT scan without evidence of pancreatic atrophy. (**B**) Post-treatment CT scan performed 2 months after ICI-DM demonstrating a significant decrease in pancreatic size

Another patient in their 70s had received nivolumab for lung cancer for 19.6 months (42 cycles) before the occurrence of ICI-DM and DKA. As shown in Fig. 2A and B, he developed marked progressive pancreatic atrophy, with a reduced pancreatic volume from 53.99 cm^3^ to 21.38 cm^3^ before and soon after ICI-DM.

Pancreatic swelling was observed in two patients (cases 5 and 10) at the time after ICI-DM diagnosis. Atrophy was subsequently observed in case 10, while case 5 had no further images due to death two months later (Fig. 1). No pancreatitis or peripancreatic fat stranding was observed in any of the cases.

## Discussion

To the best of our knowledge, this is the first academic single-institution case series for ICI-DM in Taiwan. Similar to prior studies in Korea [19] and Japan [20], which reported incidences 0.7% and 0.3%, respectively, our study estimated the incidence of ICI-DM to be 0.35%, lower than that reported in the US (1.27%) [1]. This low incidence is comparable to that of classic T1DM in East Asia, where the documented incidence is < 5 in every 100,000 people, compared to 39.9 per 100,000 in European and other Western countries [21]. In our series, ICI-DM presented with acutely marked hyperglycemia or life-threatening DKA, and random blood glucose levels measured in the weeks preceding ICI-DM were mostly normal or slightly elevated, confirming acute hyperglycemia. Despite the relatively low prevalence of pancreatic autoantibodies, with only one patient testing positive for GAD-Ab, significant and irreversible pancreatic atrophy was observed on CT imaging in most patients after the onset of ICI-DM. These findings highlight the contrasting immunological features of ICI-DM and classic T1DM, despite a similar volume loss of previously normal pancreatic parenchyma.

The classification of ICI-DM is based on clinical and biological features and their potential pathophysiology. Four types have been identified: acute autoimmune insulin-dependent DM, type 2 diabetes-like phenotype, diabetes induced by autoimmune pancreatitis, and diabetes following autoimmune lipoatrophy [22–24]. The most commonly reported type is acute autoimmune insulin-dependent DM. In our study, we found that ICI-DM was characterized by DKA with low to undetectable levels of C-peptide and relatively low levels of HbA1c, and most patients were negative for islet-related autoantibodies. It has been reported that approximately 42 − 50% of patients with ICI-DM have at least one positive autoantibody, including anti-GAD, anti-IA-2, anti-ZnT8, or IAA [7, 16, 25]; however, the positive rate appears to be much lower than that of classical T1DM, where GAD-Ab is positive at diagnosis in 80% of cases [9, 10]. In our case series, only one of ten patients tested positive for pancreatic autoantibodies, and three of these patients remained negative for follow-up autoantibodies one year after the diagnosis of ICI-DM [26]. Stamatoulo et al. found that among three patients with ICI-DM, one had islet autoantibodies prior to ICI-DM, another had seroconversion from negative to positive after the initiation of ICI therapy, while the other patient remained negative for anti-GAD, anti-IA2 and anti-ZnT8 before and after the onset of ICI-DM [27]. Unlike classic T1DM, where the presence of autoantibodies is a reliable predictor of immune tolerance and clinical manifestation, anti-IA2 usually precedes the clinical onset of T1DM due to autoimmunity and remains the best predictor of immune tolerance and clinical manifestation of T1DM. Thus, in ICI-DM, pancreatic autoantibodies are unlikely to play a primary role in the genesis of pancreatic damage and baseline pancreatic autoantibody testing may not be particularly useful as a biomarker for predicting individual susceptibility to ICI-DM. Furthermore, race may also contribute to the difference in autoantibody prevalence, with studies in Asia showing a lower prevalence of GAD-Ab in newly diagnosed patients with T1DM compared to people from European countries [21, 28–31]. Therefore, baseline pancreatic autoantibody testing may not be useful for predicting susceptibility to ICI-DM.

Furthermore, ICI-DM shares similarities with FT1DM, a rare subtype of diabetes that is more common in East Asia, accounting for 19.4% of T1DM in the Japanese national survey [32]. The clinical characteristics of FT1DM are characterized by acute onset diabetes with extremely rapid progression of β-cell destruction, severe ketoacidosis, near-normal HbA1c levels, rapid decline in C-peptide level, absent autoantibodies, and elevated pancreatic enzyme levels [33]. In 98% of FT1DM, lipase and/or amylase are increased, indicating the involvement of exocrine pancreatic cells in this type of autoimmune DM [34]. However, the etiology and pathogenesis of FT1DM remain unclear, but are thought to be associated with genetic, environmental (viral infection), and autoimmune factors; although FT1DM was considered to be unrelated to autoimmunity because diabetes autoantibodies were negative [35].

Notably, in our study, 20.0% (1/5) of patients with ICI-DM had elevated lipase levels upon presentation, which is lower than reported in other studies (52–69%) [7, 36]. However, the elevated pancreatic enzyme levels observed in both FT1DM and ICI-DM may indicate the onset of fulminant DKA, suggesting that clinicians should monitor pancreatic enzymes and volumes (via imaging), as well as blood glucose levels in patients receiving ICIs. Table 3 compares the characteristics of various types of DM and ICI-DM with those of our patients.

**Table 3:** The characteristics of the various types of DM and ICI-DM

	FT1DM [7]	Classic T1D [8]	ICI-induced diabetes [25]	Our study
Clinical features				
Ethnicity	Asian	Mostly non-Asian	Both Asian and non-Asian	Taiwan
Honeymoon	No	68.9% (71/103) [37]	No	No
Ketoacidosis	100.0%	38.8% (285/735) [38]	67.5% (135/200)	60.0% (6/10)
Permanent insulin required	Yes	Yes	98% (152/155)	100% (10/10)
Biochemical features (at diagnosis)				
C-peptide	Low or undetectable	Progressive decline, 48% maintain stimulated C-peptide > 0.2nmol/L at 5 years	63% (83/131) low or undetectable	Low or undetectable: 100% (10/10)
HbA1c (mmol/mol) (range)	46 (37–56) [39]	> 46 [40]	62 (40–120)	72 (46–101)
Lipase elevation	85% (50/59) [39]	24% (36/150) [41]	52% (13/25) [7]	20% (1/5)
Anti-GAD autoantibodies	5% (7/145) [39]	89% (114/128) [39]	43% (65/151)	10% (1/10)
Pancreas image	Undiminished pancreatic volume [42]	Pancreatic volume decreased compared with controls [43]	11% (4/36) with pancreatic atrophy [25]	90% (9/10) with pancreatic atrophy

Abbreviations: FT1DM, fulminant type 1 diabetes mellitus; T1D, type 1 diabetes mellitus; ICI, Immune checkpoint inhibitor; HbA1c, glycated hemoglobulin; Ab, antibody; Anti-GAD, Glutamic acid decarboxylase antibodies

Several studies have observed pancreatic atrophy and loss of insulin secretory function have been observed in patients with T2DM and classic T1DM [44–46]. Some investigations into pancreatic size in T1DM have also described pancreatic atrophy before the clinical disease onset, suggesting that reduced pancreatic volume may be an early biomarker of T1DM [47]. Pancreatic inflammation has also been detected at the onset of non-ICI-related FT1DM [48]. In the case of ICI-DM, rapid onset is often reflected by the loss of previously normal pancreatic parenchymal volume. However, studies have found variable pancreatic volume changes in ICI-DM, including progressive pancreatic atrophy, pancreatic enlargement, pancreatic inflammation, and no change at all. Our study found that most patients experienced a significant reduction to pancreatic volume from baseline after ICI-DM diagnosis (median: -19.92%) and at long-term follow-up (median: -37.14%), with two patients showing slight swelling of the pancreas immediately after the diagnosis of ICI-DM and one subsequently had atrophy, and one died. Two case series demonstrated that patients experienced a considerable reduction in pancreatic volume after being diagnosed with ICI-DM, with a median loss of 31 − 41% [16, 36]. However, one large review article demonstrated the majority of ICI-DM had a normal or enlarged pancreatic volume, and only 11.1% (4 over 36) of case had pancreatic atrophy at onset [25]. On the other hand, a heterogeneous pattern of pancreatic volume changes was observed prior to the onset of ICI-DM, with 25% experiencing a volume gain exceeding 10%, with subsequent atrophy in all patients after ICI-DM [36]. As such, fluctuations in pancreatic volumes with new or worsening hyperglycemia may herald the onset of ICI-DM.

The findings in this study have some limitations. First, the study was retrospective in nature, and the number of patients included was small. Second, human leukocyte antigen (HLA) genotyping was not performed. In addition to autoantibodies, certain high-risk HLA types are associated with increased susceptibility to T1DM [22]. As cell mass is thought to comprise 1–2% of the pancreas, suggesting that both endocrine and exocrine compartments change because of ICI-DM, although exocrine dysfunction has not been clinically described in this patient population. Finally, autoimmune thyroid toxicity from ICI may serve as a biomarker for antitumor immune responses, as an overly robust immune system could result in increased antitumor efficacy [49, 50]. Further evaluation of this association between ICI-DM and antitumor efficacy would be of great interest.

## Conclusions

With the increasing use of ICIs, the incidence of ICI-DM is expected to increase, causing growing concern among clinicians. In this report, we present ten cases of ICI-DM, a potentially unique form of insulin-dependent autoimmune diabetes that shares similarities and differences with autoimmune diabetes. Relying solely on pancreatic autoantibody testing is insufficient to diagnose ICI-DM. Instead, conventional CT radiographs assessing pancreatic volumes during treatment may be a significant subclinical aspect of ICI-DM development. Our findings highlight the association between pancreatic volume decline and ICI-DM and provide valuable insights for preventing endocrine emergencies.

### Electronic supplementary material

Below is the link to the electronic supplementary material.

Supplementary Figure 1: Serial random blood glucose monitoring was conducted for Case 6, with levels indicated by the black line (mmol/L) above each black dot, and the HbA1c levels, represented by the gray line (mmol/mol) above each gray dot. At 19 weeks after ICI initiation, she experienced a sudden onset of symptoms and presented with DKA.

## Data Availability

The datasets generated AND analyzed during the current study are not publicly available due to IRB regulation but are available from the corresponding author on reasonable request.

## Abbreviations

ICI: Immune checkpoint inhibitor
DM: diabetes mellitus
DKA: diabetic ketoacidosis
FT1DM: fulminant type 1 diabetes mellitus
CTLA-4: cytotoxic T-lymphocyte antigen 4
PD-1: programmed cell death 1
PD-L1: programmed cell death 1 ligand
irAE: immune-related adverse event
HbA1c: glycated hemoglobin
CT: computed tomography
MRI: magnetic resonance imaging
T1DM: type 1 diabetes mellitus
T2DM: type 2 diabetes mellitus
GAD: glutamic acid decarboxylase
Ab: antibody
IA2: islet antigen 2
ZnT8: zinc transporter 8
IAA: insulin autoantibody
ELISA: enzyme-linked immunosorbent assay
RECIST: Response Evaluation Criteria in Solid Tumors
MDI: Multiple daily injections of insulin
HLA: human leukocyte antigen
BMI: body mass index
