# Supplementary figures and images for: Distinct changes to pancreatic volume rather than pancreatic autoantibody positivity: insights into immune checkpoint inhibitors induced diabetes mellitus
Source: Diabetol Metab Syndr. 2024 Jan 23;16:26. doi: 10.1186/s13098-024-01263-6 (PMC10804587; doi:10.1186/s13098-024-01263-6)

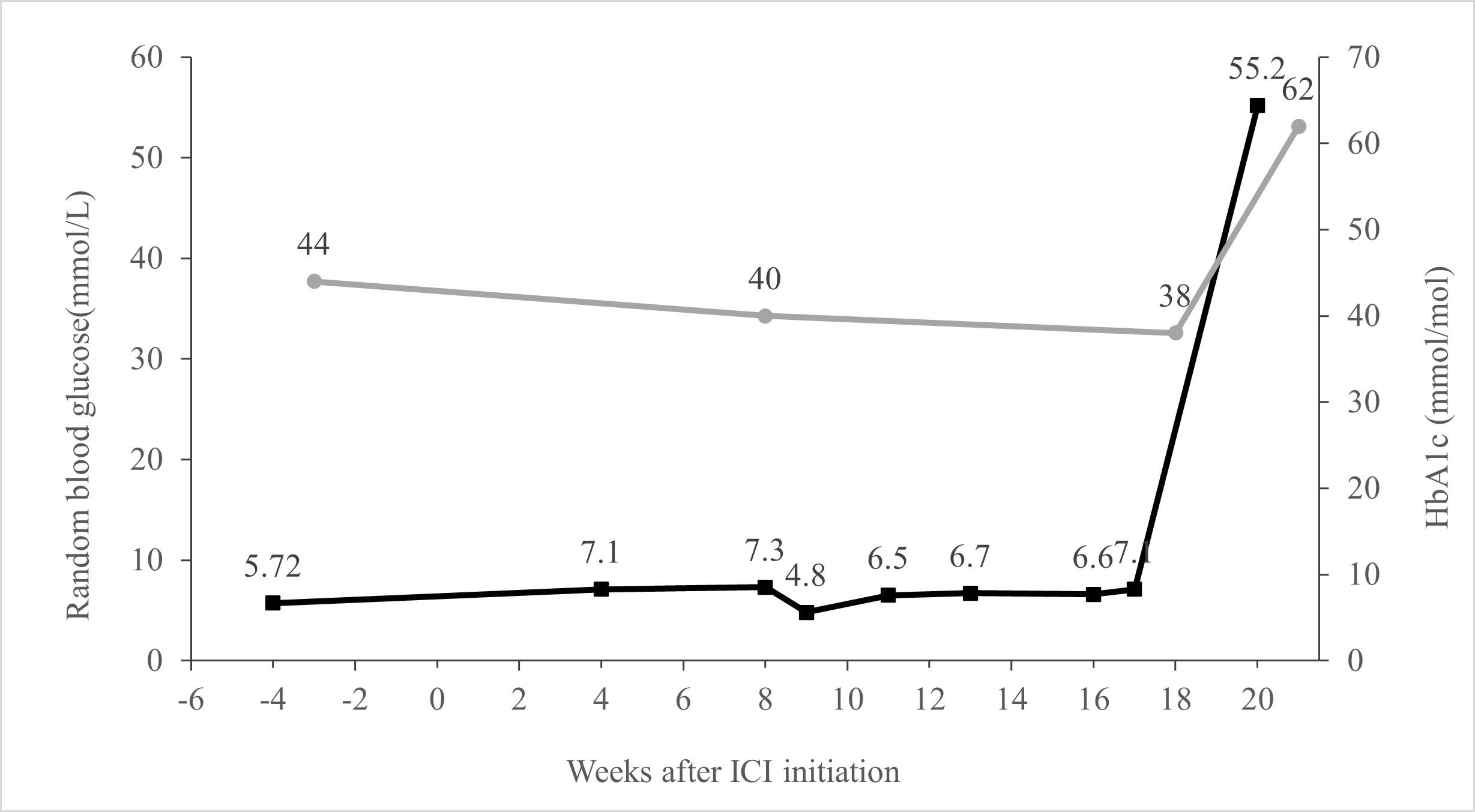

Supplement: Supplementary file 1 — Supplementary Figure 1: Serial random blood glucose monitoring was conducted for Case 6, with levels indicated by the black line (mmol/L) above each black dot, and the HbA1c levels, represented by the gray line (mmol/mol) above each gray dot. At 19 weeks after ICI initiation, she experienced a sudden onset of symptoms and presented with DKA. [file 13098_2024_1263_MOESM1_ESM.jpg]
